# Supplementary material for: Are carbon emissions trading and green financial instruments synergistic? -Comprehensive quantitative research based on content analysis
Source: PLoS One. 2024 Mar 7;19(3):e0298601. doi: 10.1371/journal.pone.0298601 (PMC10919601; doi:10.1371/journal.pone.0298601)
Supplement: S1 Data — (ZIP) [file pone.0298601.s001.zip › Data/地级市Z_F工作报告.zip!/║╙─╧/╓▄┐┌╩╨/└┤╫╘╣1⁄2╓┌║┼í╛┬φ┐╦╩2╛▌═°í┐_2007.docx]

各位代表：

　　现在，我代表市人民政府，向大会作政府工作报告，请予审议，并请各位政协委员和其他列席人员提出意见。

　　一、一届政府工作回顾

　　市一届人大一次会议以来，市人民政府在中共周口市委领导下，以邓小平理论和“三个代表”重要思想为指导，认真贯彻落实科学发展观，团结带领全市人民，艰苦创业，开拓进取，扎实工作，基本完成市一届人大历次会议确定的目标任务。

　　综合经济实力不断增强。2006年全市生产总值677.7亿元，是2000年的1.6倍，年均增长8.9%；一、二、三产业分别实现增加值225亿元、278.1亿元和174.6亿元，分别是2000年的1.4倍、2.1倍和1.9倍，年均增长5.5%、13.2%和10.8%。二、三产业增加值占生产总值的比重达到66.8%，比2000年提高6个百分点。人均生产总值6313元，是2000年的1.5倍，年均增长8.1%。地方财政一般预算收入15.2亿元，是2000年的2.2倍，年均增长12.2%。全部工业增加值244.4亿元，是2000年的2.2倍，年均增长14%。粮食总产量达到130亿斤，单产和总产均创历史最高水平。全社会消费品零售总额237.4亿元，是2000年的2倍，年均增长12.4%。

　　基础设施建设步伐加快。全社会固定资产投资累计完成1059.4亿元，年均增长24.6%。南洛、大广、周商3条高速公路周口段建成通车，许亳高速周口段即将竣工，8个县市区通达高速，高速通车里程达到334.5公里。干线公路通车里程1423公里，农村公路通车里程9512公里。高速、干线和农村公路总里程达到11269.5公里。漯阜铁路周口段完成升级改造，沙颍河实现季节性通航，公路、铁路、水路三位一体的大交通格局基本形成。沙颍河、涡河周口段近期治理工程先后开工，一批农田水利项目相继投用，防洪除涝能力明显提高，连续两年夺得省“红旗渠精神杯”。城乡面貌发生明显变化。城镇基础设施累计完成投资79亿元，城镇建成区面积由2000年的100平方公里增加到250平方公里，城镇人口由103万人增加到225.2万人。中心城市老城区改造、开发区建设和东新区开发同步进行，城市框架逐步拉大。交通路、环城路、沙南污水处理厂、第三水厂、天然气利用工程等一批基础设施项目建成使用，城市功能日益完善。县城基础设施建设和旧城改造迈出较大步伐，小城镇建设水平明显提高，城镇面貌显著改观，城镇化率达到21%。发展沼气用户40多万户，建成生态文明村210个。80%的行政村实现了村村通油路，乡镇全部通班车。改革开放逐步深入。莲花味精、周口纱厂等重点企业改制进展顺利，98%的国有工业企业完成改制任务，累计盘活国有资产49亿元。非公有制企业占规模以上工业企业总量的63%，非公有制经济占GDP的比重达到56.2%。全部免征农业税、落实惠农政策，全市农民累计减负增收23.4亿元。完成粮食流通体制改革、农村信用社改革和新一轮市县乡政府机构改革，发展活力明显增强。对外开放不断扩大，累计完成进出口总额12.5亿美元，年均增长23.8%。61家外资企业落户周口，实际利用外资1.9亿美元，年均增长38.6%。周口海关正式开关运行，市经济开发区顺利通过国家批准。上年劳务输出242万人次，劳务总收入110亿元，分别是2000年的2倍和3倍。人民生活水平得到提高。在职职工人均工资9237元，城镇居民人均可支配收入7218元，农民人均纯收入2641元，分别是2000年的1.9倍、2倍和1.4倍，年均增长分别为11.4%、12.1%和5.5%。城乡居民储蓄存款余额437.9亿元，是2000年的2.3倍。开行周口至北京和广州的旅客列车，方便了群众出行。在太康县和项城市开展新型农村合作医疗试点，参合农民163.8万人，参合率77%。解决448个村、71.7万人的饮水安全问题。全面完成城市和农村电网改造，实现城乡居民生活用电同价。累计新增城镇就业人员31.8万人，下岗失业人员再就业14.7万人，城镇登记失业率控制在4.5%以下。国有企业下岗职工基本生活费、失业人员失业保险金、企业离退休人员养老金按时足额发放，城乡困难群众基本生活得到有效保障。社会事业全面进步。相继建成莲花味精、金丹乳酸和辅仁药业3个博士后工作站，取得市级以上科研成果419项。圆满完成农村中小学D级危房改造任务。民办教育成为全省亮点，高考成绩居全省先进行列。周口师范学院实现专升本。周口职业技术学院实现5校区实质性合并，办学质量明显提高。

　　夺取抗击“非典”胜利，建成市县10个疾病预防控制中心、市传染病医院、市紧急救援中心和8个县级传染病区等一批公共卫生项目，疾病预防控制和医疗救治体系进一步完善。成功举办两届中华姓氏文化节和弘扬老子文化国际研讨会，我市被命名为中国“杂技之乡”、“三皇故都文化之乡”和“神话学研究基地”。竞技体育水平不断提高，在省级以上比赛中获得金牌204枚。人口和计划生育工作赶上全省先进水平，10个县市区全部达到二类县以上标准。大力开展土地“三项整治”，新增耕地7240公顷，实现土地占补平衡。进一步加大污染治理力度，累计关闭、取缔污染严重的企业316家，6个城市污水处理项目建成试运营，2个县市垃圾处理厂开工建设。安全生产形势逐步好转，事故起数和死亡人数逐年下降。审计、统计、物价、监察、广播电视、民政、外事侨务、爱国卫生、气象、人防、民兵预备役、档案、史志等各项社会事业取得了新的成绩。

　　精神文明和民主法制建设进一步加强。深入开展“五城联创”活动，积极开展向陈新庄、洪战辉等先进人物学习活动，扎实开展“双百”评选活动，城乡文明程度不断提高。顺利完成第四和第五届村委会换届选举，政务公开、厂务公开和村务公开不断扩大。完成“四五”普法任务，法治政府建设和依法行政工作扎实推进。自觉接受人大法律监督、工作监督和政协民主监督，累计办理人大代表建议572件和政协委员提案1373件。密切联系工会、共青团、妇联等人民团体，广泛听取社会各界意见和建议。加强政府廉政建设，反腐败斗争取得新成效。深入开展双拥工作，荣获省级“双拥模范城”称号，项城企业军转干部解困稳定工作经验得到中央肯定。加强社会治安防控体系建设，命案攻坚、打黑除恶、打击遏制“两抢一盗”等专项斗争取得显著成效。深入开展民族团结进步教育活动，民族宗教工作得到进一步加强。依法做好信访工作，建立健全应急机制，社会大局保持稳定。各位代表，回顾六年发展历程，总结六年工作实践，主要体会是“五个必须坚持”：一是必须坚持科学发展观和正确政绩观，始终把发展作为第一要务，把科学发展作为根本原则，注重转变经济增长方式，注重优先发展工业，注重项目建设和招商引资，走符合周口市情的发展道路。二是必须坚持解放思想、实事求是的工作方法，不主观主义，不急功近利，不盲目瞎干，一切从实际出发，真抓实干，注重实效，着力解决好发展中的矛盾和问题，推动经济社会协调发展。三是必须坚持以人为本、执政为民，把人民群众利益放在首位，尽力多办些攸关民生的实事、好事，多解决一些群众反映强烈的热点、难点问题，让人民共享改革发展成果。四是必须坚持团结奋进、齐心协力，充分调动方方面面的积极性，凝聚广大干部群众的智慧和力量，形成方向明确、思想统一、加快发展的强大合力，树立周口的良好形象。五是必须坚持优化发展环境，始终把强化服务、提升效能、讲究诚信摆在突出位置，着力打造加快发展的平台，不断增强我市的竞争力。这些体会，既有经验也有教训，是六年工作实践的探索和总结，也是今后我们应该继续遵循和把握的基本原则。各位代表，过去六年取得的成绩，是省委、省政府和市委正确领导的结果，是市人大、市政协大力支持的结果，是全市人民共同奋斗的结果。在此，我代表市人民政府，向工作在全市各条战线上的工人、农民、知识分子、干部、人民解放军指战员、武警消防官兵、公安干警，向各民主党派、工商联、无党派爱国人士、离退休老同志和各界人士，致以崇高的敬意！向驻周单位及所有关心、支持我市现代化建设的同志们、朋友们，表示衷心的感谢！在肯定成绩的同时，我们也要清醒地认识到，我市人口多，基础差，底子薄，结构性矛盾突出，人均发展水平低。经济社会发展中还存在着一些困难和问题，主要是：工业发展缓慢，骨干企业弱，中小企业少，新上项目不多，发展后劲不足；农业基础依然薄弱，粮食稳定增产、农民持续增收难度加大；城市化水平偏低，中心城市集聚辐射带动效应不强；县域经济整体发展水平不高，县乡债务包袱沉重，财政收支矛盾突出；上学难、看病贵问题反映强烈，部分群众生活困难；部门服务意识、办事效率和依法行政能力有待加强；吃拿卡要、强装强卸、不讲诚信的现象常有发生，经济发展环境仍不宽松。对这些问题，我们一定要高度重视，采取更加有力的措施，在今后的工作中切实加以解决。

　　二、今后五年总体要求和奋斗目标

　　各位代表，今后五年是周口经济社会发展的关键时期，我们必须正确认识目前面临的形势。“十五”期间，我市经济总量是全省18个省辖市中，唯一一个呈1位数增长的市，财政收入、城乡居民收入、工业增加值等主要经济指标居于全省落后位置。2005年全省县域经济发展综合实力排序，我市9个县市，7个排位下降，4个处于90位以后。整体上看，我市与全省的发展差距不是在逐年缩小，而是在逐年拉大，经济总量在全省的比重下降。经济发展处于工业化的初期阶段、城镇化的起步阶段和传统农业向现代农业的转变阶段。面对这样的局面，我们压力空前，责任重大。如再不警醒，奋起直追，尽快改变落后面貌，就会拖全省发展的后腿。各位代表，我们既要正视落后的现实，又要看到我市加快发展的五个有利条件：一是具有加快发展的宏观环境。经济全球化继续深入发展，科技进步日新月异，国际国内产业转移步伐加快，世界经济正处于新一轮增长期，宏观经济形势对我市发展较为有利。二是具有加快发展的难得机遇。中央更加重视“三农”工作，出台了一系列支农惠农政策，对中部贫困地区和革命老区，比照西部大开发有关政策给予支持，我市7个县可比照西部大开发享受有关优惠政策。最近在我市召开的全省部分市县经济发展座谈会上，省政府明确表示，要对省内黄淮经济区加大扶持力度。三是具有加快发展的一定基础。经过历届班子和全市人民的共同努力，我市经济总量达到一定规模，综合实力明显提升，内在活力正在形成。四是具有加快发展的广阔空间。农副产品资源和劳动力资源丰富，发展农副产品加工业和劳动密集型制造业前景广阔，为扩大开放、招商引资提供了有利条件。五是具有加快发展的浓厚氛围。市二次党代会明确提出了“三个转变”、“两大建设”和“一个奋斗目标”，为我市加快发展指明了方向，理清了思路，绘就了蓝图。全市上下政通人和，广大干部群众思发展、谋发展、求发展的热情高涨、信心倍增，团结奋进、干事创业、竞相发展的局面逐步形成。利用好这些有利条件，我市就会迎来一个大的发展。各位代表，我们一定要正确认识我市前进中存在的困难和问题，知耻而后勇；一定要紧紧抓住发展中面临的机遇和优势，迎接新挑战。牢固树立三种意识，勇于担当起历史赋予的重任。一定要树立强烈的忧患意识，增强加快发展的责任感和紧迫感，解放思想，与时俱进，把建设富裕文明和谐新周口，作为现阶段最大的政治、最硬的道理、最紧迫的任务；一定要树立强烈的机遇意识，增强抢抓机遇的能力，敏锐地发现机遇，果断地抓住机遇，创造性地利用好机遇，真正把机遇变成推动发展的强大动力和实际成果；一定要树立强烈的发展意识，聚精会神搞建设，一心一意谋发展，努力实现改革开放的新突破、经济发展的新跨越、社会文明的新进步。按照市二次党代会精神，今后五年政府工作的总体要求是：以邓小平理论和“三个代表”重要思想为指导，全面贯彻落实科学发展观，构建社会主义和谐社会，坚持又好又快发展方向，以工业化、城镇化和农业现代化为支撑，以加大投资和项目建设为着力点，以加快产业培育和集聚为突破口，着力调整经济结构和转变增长方式，着力加强资源节约和环境保护，着力推进改革开放和自主创新，着力促进社会发展和解决民生问题，努力实现追赶型、跨越式发展。按照这一总体要求，结合“十一五”规划纲要，今后五年的奋斗目标是：逐步缩小主要经济指标与全省平均水平的差距，提高经济总量占全省的比重，努力建设繁荣富裕、文明开放、稳定和谐的新周口。

　　——加快经济发展方面：到2011年，全市生产总值达到1200亿元，年均增长12%；二、三产业比重达到80%；财政一般预算收入达到28亿元，年均增长13%。

　　——推进城乡发展方面：中心城市规模进一步扩大，市区建成区面积达到70平方公里以上，人口超过65万。周商、周淮一体化建设取得一定进展。县域经济综合实力明显提升。县市城区功能逐步完善，形成一批特色乡镇。2011年城镇化率达到35%以上。

　　——坚持可持续发展方面：人口自然增长率控制在6‰以内；科技进步对经济增长的贡献率达到39%；万元生产总值能耗在2006年的基础上降低18%；全市林木覆盖率达到26%以上，市区建成区绿化覆盖率达到40%以上；出境断面水质达标率91%，市区空气质量优良天数超过295天；市区生活垃圾无害化处理率达到85%。

　　——改善人民生活方面：城镇居民人均可支配收入和农民人均纯收入，年均分别增长8%和6%；城镇新增就业人员25万人，城镇登记失业率控制在4.5%以内；城镇人口基本养老覆盖人数达到29.7万人、城镇职工参加医疗保险人数和失业保险参保人数均达到41万人；农村新型合作医疗覆盖率达到90%；形成以最低生活保障为基础、社会捐助为补充、专项救助相配套，覆盖城乡的新型社会救助体系。

　　为实现上述目标，我们必须加快三个转变和三个推进。

　　——加快工业弱市向工业强市转变。工业化是现代化的前提，也是提升农业、壮大服务业的内在动力和物质基础。周口落后在工业，潜力也在工业。必须坚持以工业为主导，实施工业兴市战略，立足农副产品资源和劳动力资源丰富的优势，着力打造农副产品加工基地和劳动密集型产业基地。以扩张总量为重点，以提高质量和效益为目的，努力走出一条传统农区工业化道路。

　　——加快农业大市向农业强市转变。我市人口80%在农村。“三农”问题是实现追赶型、跨越式发展首要解决的问题，任何时候都要重视和加强农业。必须坚持农业基础地位，以建设粮食生产核心区为重点，以加强基础设施建设为支撑，以产业化经营为途径，以提高农民素质为关键，以发展现代农业为方向，以富裕农民为根本，促进粮食稳定增产、农业显著增效、农民持续增收、农村更加和谐，扎扎实实建设社会主义新农村。

　　——加快文化资源大市向文化产业强市转变。文化旅游产业不仅是推动经济社会发展的动力，也是经济社会发展的重要组成部分。文化底蕴深厚、旅游资源丰富，是我市的优势所在。必须坚持一手抓传统文化产业的振兴，一手抓现代文化旅游产业的开发，用市场模式催生、培育、壮大文化旅游产业，提高文化旅游产业竞争力，使文化旅游产业成为我市的新兴产业。

　　——加快推进城乡统筹发展。城市是现代文明的重要载体，城市化是工业化和现代化的必然要求。必须坚持走产业兴市的道路，积极引导和推动产业向园区集中，园区向城镇集中，人口向城镇有序转移。坚持统筹城乡发展，以特色经济为依托、非公有制经济为重点，大力发展县域经济，增强县域经济综合实力，努力实现县域经济综合实力在全省位次前移。

　　——加快推进改革开放。体制优势是最具竞争力的优势，体制保障是最根本的保障。加快周口追赶型、跨越式发展，必须实现改革开放的新突破。按照完善社会主义市场经济体制的要求，加大改革攻坚力度，力争在一些重要领域和关键环节取得实质性进展。坚定不移地实施开放带动战略，不断提高开放水平。努力把深化改革和对外开放紧密结合起来，实现改革开放相互促进、双向推动，加快建设更具活力和更加开放的经济体系。

　　——加快推进和谐周口建设。构建社会主义和谐社会是推进经济社会发展的重要目标，也是经济社会发展的重要保障。必须坚持以人为本，注重社会公平，正确反映和兼顾不同方面群众的利益，正确处理改革发展稳定的关系，不断满足人民群众日益增长的物质文化需要，确保人民群众安居乐业。

　　各位代表，在实现周口追赶型、跨越式发展的新阶段，我们提出上述奋斗目标和发展思路，是在全面分析形势、认真研究市情市力基础上确立的，通过全市人民共同努力是一定能够实现的。迈上这样一个新台阶，全市综合实力将大为增强，人民群众将得到更多实惠，全面建设小康社会将取得显著成效。

　　三、2007年主要工作任务

　　今年是贯彻落实市二次党代会精神的第一年，也是新一届政府的开局之年。做好今年的工作，具有特殊重要的意义。今年全市经济社会发展的预期目标是：生产总值增长12%；财政一般预算收入增长14%；全社会固定资产投资增长18%；社会消费品零售总额增长13%；外贸出口增长16%，实际利用外资增长20%；万元生产总值能耗降低3.6%左右；化学需氧量、二氧化硫排放量分别减少2%和1%；城镇居民人均可支配收入增长8%，农民人均纯收入增长6%；居民消费价格总水平涨幅在3%以内；城镇新增就业6万人，城镇登记失业率控制在4.5%以内；人口自然增长率控制在6‰以内。这些目标中，节能降耗和污染减排指标是约束性的，必须千方百计确保完成；经济增长等指标虽然是预期性的、富民指标虽然是导向性的，但事关全局，事关发展，要力争完成得更好。实现上述目标，要突出抓好“双十五工程”，扎实推进七项重点工作：突出抓好“双十五工程”实现经济持续快速发展，项目是关键，投资是保证，必须千方百计抓好项目建设；发展经济的目的是为了人民，发展的成果要惠及人民，必须关注民生，重视民生，保障民生，改善民生，切实把好事办好、实事做实。

　　——突出抓好十五项重大项目。今年我市筛选重点建设项目110个，总投资174亿元，本年计划投资80亿元，其中，亿元以上项目38个。在整体推进的同时，着重抓好以下15项重大项目：1.总投资39.7亿元，今年完成15.8亿元，建成许亳高速公路周口段；2.总投资2.7亿元，今年完成1亿元，建成河南金丹乳酸有限公司5万吨L-乳酸工程项目；3.总投资2.12亿元，今年完成全部投资，建成辅仁药业集团年产5亿支医药、保健口服液项目；4.总投资1.96亿元，今年完成7620万元，建成河南志元食品有限公司年产5万吨熟食制品生产项目；5.总投资1.9亿元，今年完成6255万元，建成周口富农饲料公司年产20万吨饲料一期工程项目；6.总投资1亿元，今年完成全部投资，建成周口隆达发电有限公司2×13.5万千瓦烟气脱硫工程项目；7.总投资1亿元，今年完成全部投资，建成国能扶沟2×1.2万千瓦生物质能发电工程项目；8.总投资12.7亿元，今年完成5亿元，继续实施沙颍河、涡河周口段近期治理工程；9.总投资3.3亿元，今年完成9400万元，继续实施河南财鑫集团有限责任公司热电联产改造项目；10.总投资3.16亿元，今年完成1.5亿元，继续实施河南力神公司玉米联合收割机制造项目；11.总投资2.18亿元，今年完成6700万元，继续实施天鹰缸套公司年产2.3万吨缸套生产项目；12.总投资5.7亿元，今年完成2亿元，开工建设河南周口宏亿木业有限公司中高密度纤维板生产项目；13.总投资2.6亿元，今年完成1.1亿元，开工建设扶沟昌茂纺织公司15万纱锭建设项目；14.总投资1.75亿元，今年完成投资6000万元，开工建设西华景兴制衣公司年加工150万件服装项目；15.总投资1亿元，今年完成5000万元，开工建设太康220千伏变电站。

　　各位代表，我市与先进地区相比，落后就落后在工业总量小和缺少大项目上。我们要缩小差距、加快发展，就必须紧紧抓住加速工业化和上大项目这个核心，始终把工业发展和大项目突破作为加快发展的第一方略，集聚一切有效资源，调动一切有利因素，全面聚焦大项目，全神贯注大项目，全力突破大项目，每年我们都要在招商引资和抓好一批投资大、产出高、富民快的大项目上下功夫，抓出成效。全面提升我市工业经济发展水平，以大项目的突破，促进工业快速发展；以工业的快速发展，推进周口经济的跨越式发展。

　　——突出抓好十五件实事。围绕市委经济工作会议上提出的十五件实事，认真细化分解，明确责任要求，加强督促检查，切实抓好落实：

　　1.改善农民基本生产生活条件。筹措资金3亿元，新建和改造农村公路1500公里，解决1050个行政村、150万人的出行难问题，实现所有行政村通油路。筹措资金1.14亿元，解决26万农村居民安全饮水问题。争取资金4.8亿元，解决无电村(户)和盲点村用电问题。筹措资金2000万元，新增农村沼气用户20万户。

　　2.巩固提高农村义务教育水平。全部免除农村义务教育阶段学生的学杂费，继续对困难家庭学生免费提供课本、补助寄宿生生活费。为全市农村中小学更新配置课桌凳。筹措资金9710万元，维修改造农村中小学校舍19.4万平方米。筹措资金5954万元，实施农村中小学现代远程教育建设工程。

　　3.加大对农民的补贴力度。积极筹措资金，继续实施对种粮农民直接补贴、农业生产资料综合直补、优质专用小麦良种补贴、专用玉米良种补贴、农村劳动力转移培训补贴、测土配方施肥补贴、农机具购置补贴，确保补贴金额高于上年。

　　4.缓解群众看病难、吃药贵问题。对18所乡镇卫生院进行全面改造，完成所有乡镇卫生院改造目标任务。继续对贫困县县医院、中医院、妇幼保健院进行改造。巩固太康县、项城市新型农村合作医疗试点成果，抓好扶沟、沈丘、鹿邑、淮阳等4个县的试点工作。做好城市社区医疗卫生服务，完善城市医疗卫生体系。继续整顿医疗服务价格，扩大按单病种限价收费试点范围，调整政府定价范围内药品价格，对价格偏高药品进行分期分批降价。

　　5.繁荣农村文化事业。在300个行政村实施农村电影放映工程。恢复50个标准乡镇文化站，建设100个村文化大院，培育并命名5个“文化产业示范乡”、20个“文化产业示范村”。

　　6.做好农村劳动力转移就业工作。继续实施农村劳动力技能就业计划、农村劳动力培训阳光工程、贫困地区劳动力转移培训雨露计划，完成引导性培训5万人、技能性培训5.5万人，全年新增15万农村富余劳动力转移就业。实施工资支付保证金制度。认真解决进城务工农民子女上学难问题。

　　7.加强城镇就业再就业和社会保障工作。全面落实就业再就业优惠政策，积极促进下岗失业人员再就业。加大对“4050”人员、城镇低保人员、零就业家庭等困难群体再就业帮扶力度，基本消除零就业家庭，实现下岗失业人员再就业3.05万人，其中“4050”人员1.1万人。扩大社会保险覆盖面，新增城镇基本养老保险参保人数1.1万人，城镇基本医疗保险参保人数达到37.8万人。

　　8.加大对城乡困难群体救助力度。完善农村低保制度，农村低保最低补差标准提高到30元。农村五保户集中供养率提高到30%。增加对城乡医疗救助的资金补助，确保补助金额高于上年。加大扶贫开发力度，力争10万农村贫困人口脱贫和解决温饱。

　　9.加快经济适用房和廉租房建设。规范经济适用房管理，新增经济适用房面积10万平方米，不断改善低收入家庭住房条件。加快廉租房工程建设，逐步解决困难群体住房难问题。

　　10.进一步加强污染防治。将沙颍河、汾河、贾鲁河、惠济河、?河、涡河作为流域水污染综合整治重点，达到省政府目标要求。重点治理化工、医药、皮革等重污染行业，对8家企业进行综合整治，形成年削减化学需氧量600吨、二氧化硫排放量300吨的能力。在全市所有县市建成污水处理厂，并建立运营的长效机制。加大市垃圾处理、医疗废物处置及县级垃圾处理工程建设力度，确保年底前全部建成。

　　11.继续抓好乡镇客运站和“万村千乡”市场工程建设。筹措资金2900万元，完成58个乡镇客运站建设，实现每个乡镇均有一个具有一定规模的客运站目标。筹措资金1000万元，新建、改建农家店2000家，推动农村市场体系建设。

　　12.继续加快完善沙颍河城区段综合开发。建成沙颍河橡胶坝，实现城区段截流蓄水。完成沙颍河城区段二期改造治理工程，两岸滨河游园投入使用。

　　13.继续加大城区基础设施建设和改造力度。抓好老城区次干道亮化工程，硬化小街小巷道路100条，解决城区居民出行难问题。新增完善社区绿地20处，增加绿化面积80万平方米。

　　14.继续推进天然气利用工程。中心城区内新铺设天然气管网40公里，实现新增用户1.5万户。确保9个县市天然气利用工程建成投用。

　　15.加快城区学校建设步伐。结合我市区划调整和中心城区改造，重新编制城区教育布局规划，合理配置教育资源，在市区新建1所小学，改扩建1所初中，逐步缓解市区学生入学难问题。

　　各位代表，人民群众是我们的衣食父母，为人民服务是党和政府的根本宗旨。我们一定要牢固树立执政为民的理念，怀着深厚的感情为群众做好事、办实事、解难事。尽管我们不能在短期内把所有问题都解决好，但我们可以从群众最急需解决的问题入手，从能够办好的事情做起，每年都办成一些与群众利益密切相关的实事、好事，让广大人民群众得到更多的实惠，共享改革发展成果。扎实推进七项重点工作：

　　(一)扎实推进工业兴市战略，突出工业主导地位坚持以新型工业化为核心，做大工业经济总量，形成工业为主导的经济结构，推进产业结构优化升级。加快产业聚集区建设。按照“布局集中、用地集约、产业集聚”的原则，科学规划产业聚集区，完善聚集区基础设施和公共服务体系，加快标准厂房建设，促进各类聚集区依托比较优势，发展特色产业，实现规模效益。重点抓好扶沟纺织工业园、项城鞋业工业园、淮阳塑编工业园、郸城玉米精深加工工业园建设，积极培育沈丘网业、鹿邑尾毛、太康锅炉、扶沟机械制造、项城农机配件等产业集群，力争成为省重点扶持的产业集群。加快市级重点扶持的产业聚集区建设，促进一批专业化水平高、配套能力强的中小企业发展，形成一批新的产业群、产业带。培育壮大龙头企业。按照“扶优、扶强、扶大”的原则，加速资本聚集，促进资源和品牌整合，形成一批知名度高和带动力强的大企业、大集团。今年重点抓好“五个一批”，即：改制盘活一批，做好莲花集团、宋河酒厂、周口纱厂等国有企业改制完善工作，帮助华林集团、周口通信电缆厂加快改制步伐，引进战略投资者，争取早日恢复生产，重现生机；改造提升一批，加快对金丹乳酸、财鑫集团、金丝猴集团等现有龙头企业的技术改造，通过低成本扩张，促进龙头企业上水平、壮实力、增后劲；培育壮大一批，整合企业资源，鼓励莲花股份、辅仁药业、鞋城皮革集团等有条件的企业通过兼并、资产重组等形式，扩大规模，争取培育一批销售收入超过30亿元和50亿元的企业；引进发展一批，发挥比较优势，切实加大招商引资力度，重点引进一批有较强实力、有知名品牌的大企业，扩大龙头企业群体；谋划储备一批，围绕八大支柱产业，认真谋划一批科技含量高、聚集效益大、产业链条长、带动能力强的项目。做大做强市本级工业。以市开发区为平台，实施灵活的政策，营造良好的环境，吸引工业项目入驻，全面提升市本级工业集聚发展水平。进一步改造激活市本级企业，加快发展中小企业，继续着力引进外资企业。重点支持益海、五得利、天瑞、亿星等企业加快发展步伐，积极推进邦杰集团、周口发电公司等企业加快改革，抓好周口骆驼饲料公司年产30万吨饲料等一批工业项目，积极培育新的经济增长点。增强自主创新能力。坚持依靠科技进步与创新，促进经济增长方式转变，发挥我市资源和现有产业优势，推广和运用现代技术，改造传统产业。加大技术改造力度，淘汰落后生产能力，带动产业结构优化升级。充分发挥企业在科技创新的主体作用，加强产学研合作，促进科技成果转化，保护知识产权。依托河南省造纸网业协会和中国造纸网科技研发中心，力争在优势产业核心技术方面取得突破。重点扶持莲花味精、辅仁药业2个国家级企业技术中心和8个省级企业技术中心的发展和完善。大力推进名牌战略，争取在莲花牌味精、宋河粮液、邦杰牌火腿肠、口福牌食用油等知名品牌的基础上，使我市有更多的产品跨入全省、全国的名牌序列。加强资源节约和生态环境保护。强化节约资源和保护环境目标责任制，确保完成节能降耗和污染减排任务。重点抓好金丝猴集团、财鑫集团、金丹乳酸公司循环经济试点工作，促进资源综合利用。全面落实项目建设环保“三同时”等各项环保制度，加大污染企业治理，限期完成整改治理任务。壮大企业家队伍。高度重视企业家在经济发展中的重要作用，坚持培养和引进相结合，不断壮大企业家队伍，提高企业家素质，引导企业家担负起社会责任。加快成立企业家协会，为企业家提供学习交流、沟通联动平台。切实关心、爱护和保护企业家，建立对有突出贡献的企业家政治上鼓励、经济上激励的长效机制。充分发挥企业家的智慧，激发企业家的创业激情，营造有利于企业家干事创业的良好环境。

　　(二)扎实推进现代农业发展，建设社会主义新农村更加重视和加强农业基础地位，以发展产业、富裕农民、改善村容为重点，统筹兼顾，量力而行，全面推进新农村建设。着力打造粮食生产核心区。抓好国家大型商品粮生产基地续建项目、国家优质粮食工程生产基地县项目。打造国家储备粮生产储备基地。高标准完成5个农业综合开发重点县和面上县20万亩以上中低产田改造任务，全面提升农业综合开发水平。搞好农田水利基本建设，开展“红旗渠精神杯”竞赛活动。认真落实各项支农惠农政策，防止农民负担反弹，保护和提高农民种粮积极性。稳定和提高粮食作物种植面积，依靠科技，改善品质，提高单产。加快推进农业结构调整。推动农业区域化布局调整，形成优势农产品产业带，提升区域竞争优势。重点支持以扶沟、商水、川汇区为主的瓜菜生产，以淮阳、川汇区为主的食用菌生产，以扶沟、西华、太康、项城为主的林果和花卉苗木种植，以鹿邑、郸城为主的中药材生产，以沈丘为主的强筋小麦生产。加强小麦良种繁育基地建设，把我市建成黄淮流域最大的良种繁育基地。加强农产品质量认证和检验检测体系建设，发展无公害农业，力争无公害农产品生产面积达到200万亩。大力发展畜牧业，积极推进规模养殖，加强畜牧防疫工作，逐步实现畜牧业标准化生产。从今年起，力争3年内中型规模养殖场和规范化养殖小区发展到1000个；加快林业生态县建设，发展林产品加工业，使其成为农民增收的亮点和途径。

　　积极推进农业产业化经营。大力发展食品加工、纺织服装、医药制造、皮革皮毛、板材加工等支柱产业，依托莲花味精、益海粮油、五得利面粉、金丹乳酸、志元食品、金丝猴集团等龙头企业，拉长农副产品加工链条，引导龙头企业在农村建基地、连农户，发展订单农业，使企业和农户形成一种长期、稳定、牢固的利益共同体。大力发展特色市场和农业生产要素市场，重点抓好扶沟县昌泰农产品物流配送中心等项目建设，搞活农产品流通。发挥市农业产业化龙头企业协会的作用，加强企业之间、银企之间的合作与交流，提升企业竞争力。积极发展农民专业合作经济组织，引导和鼓励广大农户在自愿的基础上，组建各种专业合作社，提高农业的组织化程度。大力发展劳务经济。充分发挥周口职业技术学院、海燕职专等100所职业技术培训学校的桥梁作用，搞好农民工培训，切实提高外出务工农民的技能水平。巩固提升“项城防水”、“西华的哥”、“沈丘集装货运”等一批在全国有影响的劳务品牌，努力打造一批新的劳务品牌。利用我市已具备国外劳务输出自主权的优势，积极扩大出国务工规模，进一步提升劳务输出层次，推动人口大市向人力资源大市、劳务输出大市向技工输出大市转变。实施回归工程，鼓励外出农民工回乡创业。今年劳务输出257万人次，切实增加劳务收入在农民收入中的比重。

　　(三)扎实推进城市化进程，统筹城乡发展坚持高起点规划、高标准建设和高效能管理，进一步拓展发展空间，改善人居环境，提升城市品位。大力发展县域经济，实现城乡统筹发展。增强中心城市承载力和辐射力。认真落实省政府批准的中心城市总体规划，按照“政府组织，专家领衔，部门配合，公众参与，科学决策”的要求，抓紧分区规划、专项规划、控制性详细规划和城市景观规划的编制和完善，用规划引领城市建设。牢牢把握“充实、完善、提高”的原则，一手抓城市资源优化配置和基础设施配套完善，突出特色，打造城市亮点；一手抓小街小巷改造，实施硬化、亮化、净化、绿化“四化”工程，改善市民居住环境。按照打造工业基地、聚集城市产业的要求，完善组装开发区。理顺管理体制，创新工作机制，优化发展环境，提升开发区的吸引力和竞争力，加大招商引资力度，力争在引进技术含量高、投资规模大、辐射带动能力强、财税贡献率高的项目上求突破。严把准入门槛，坚持走节约型、环保型、可持续发展的道路，禁止投资规模小、产业层次低、技术含量低、经济效益低的项目入驻，切实提高土地利用率，努力把开发区打造成我市招商引资的平台、对外开放的窗口、工业发展的基地、市本级的重要财源。抓好南外环、沙南污水处理厂等配套基础设施建设，尽快启动已具备条件、符合产业要求和规划布局的项目。建成并投入使用市综合办公楼和市财政局、教育局、建委、国土资源局等单位的办公楼，切实改善市直单位的办公条件。坚持以人为本、量力而行、尽力而为，改造提升老城区。完善城市功能，打通部分街道断头路，加强通讯、供水、供气、供暖、照明等基础设施建设，及时清除小街小巷居民生活垃圾，加快沙北老城区改造，努力改善市民的居住生活环境。切实抓好关帝庙商业区建设、新世纪半岛花园建设和西大街、育新街旧城改造。以项目建设为重点，开发建设东新区。把东新区建设作为拉大中心城市框架、加快周淮一体化进程的现实选择，进一步加快道路、供排水管网等市政基础设施建设，完善功能，提升吸纳能力。抓好市广电中心、体育馆、报业大厦、公务员小区、市委党校和周口一高新校区等项目的实施，加快东新区组装步伐，争取早日见成效、成规模。统一城乡规划和管理，推进周商一体化、周淮一体化。把川汇区、淮阳、商水作为中心城市建设的重要支撑，合理布局，统筹发展。建立“一体化”规划与建设联席会议制度，有关县区明确专人负责，切实做好衔接和协调工作，科学配置有效资源。大力发展城市经济。立足现有城市生产力布局，搞好资源整合。遵循产业聚集规律，培育城市产业。在中心城市西北角加快发展以发电、水泥、建材工业为主的重工业；在市区南面搞好粮油、食品加工业的聚集和发展。大力发展以服务业为主的城市特色产业，改造提升商贸流通、交通运输等传统服务产业，加快发展中介服务、社区服务等新兴服务业。积极发展商贸物流业，抓好荷花市场、中原国际商贸城等一批有特色、辐射能力强的市场，形成城市发展的市场支撑。着力发展建筑业和房地产业，扩大经济适用房和廉租房建筑规模，年新增经济适用房建筑面积不低于商品房竣工面积的10%，逐步改善低收入家庭住房条件。

　　强化城市管理。完善“两级政府、三级管理、四级网络”的城市管理机制，充分发挥川汇区、开发区的作用，加大社区管理力度，推动管理重心下移和管理工作下延，把城市管理延伸到居民小区和小街小巷，切实做到统一领导、属地管理、部门协调、权责分明。加强城市精细化管理，建设宜居城市。以“五城联创”为载体，着力解决城市违章搭建、占道经营、交通秩序混乱、乱倒垃圾等难点问题，整治市容市貌，搞好美化亮化，建立健全城市管理长效机制。

　　搞好县城和小城镇建设。加强城镇的规划、建设和管理，多元投资，加快县城和小城镇建设步伐。突出抓好国家、省确定的鹿邑玄武、项城秣陵等38个试点镇、重点镇的建设和发展，坚持把县城和小城镇建设与市场建设、产业集聚发展结合起来，增强城镇综合经济实力和辐射力，引导人口向城镇发展，产业向园区集中，以人口和产业集聚带动县城规模扩大和中心城镇发展。深化户籍管理制度改革，为农民进城创业、就业、定居营造良好的社会环境。

　　加快发展县域经济。县域经济是统筹城乡发展的重要结合点，要以促进农民增收、增加财政收入、壮大经济实力为主要任务，大力发展县域经济。充分发挥各自比较优势，转变发展方式，拓宽发展思路，把优势产业做出特色，把特色产业做出规模，形成特色鲜明的县域经济发展格局。项城、鹿邑、沈丘、郸城要发挥工业基础较好的优势，加快调整经济结构，提升产业层次和区域竞争力。西华、扶沟、太康要在发展现代农业的同时，加快工业化、城镇化进程，不断增强自我发展能力。围绕产业化龙头企业和城市经济发展配套产业，推动产业集群发展。扩大招商引资，吸引更多的外来创业主体，解决县域生产要素有限、自身积累不足的问题，以内部资源嫁接外部资源，以外生力量激活内生力量。坚决贯彻中央和省促进县域经济发展的方针政策，加大扶持力度，为县域经济发展创造良好环境。

　　(四)扎实推进文化旅游产业发展，培育新兴产业抓住国家及我省发展文化旅游产业的机遇，把我市文化旅游资源优势转化为经济优势，形成新的经济增长点。大力发展文化产业。围绕我市“伏羲文化、老子文化、姓氏文化、农耕文化”四种文化资源，着力打好“三皇故都、道家之源、姓氏之根、杂技之乡”四张文化牌，大力发展以文化资源开发、文化产品经营、文化设施利用为主的文化产业。积极筹办首届国际老子文化节，做好第三届姓氏文化节筹备工作。重视周口越调、太康道情、太昊伏羲祭典和老子生日祭典等非物质文化遗产的保护和发展。整合书画经营、艺术培训、杂技培训与演出等文化资源，培育新的经济增长点。依托周口日报社、周口电台、电视台等现有资源，培育一批市场占有率高、品牌响、扩张能力强的文化产业集团，切实增强文化产业的竞争力。大力发展旅游产业。围绕创建“全国优秀旅游城市”，搞好旅游发展总体规划。大力实施旅游精品战略，完善景区配套设施，以“周—淮—鹿”三点一线旅游带为重点，抓好太昊陵、老子故里等综合开发项目，努力把太昊陵景区、老子故里旅游区建成国家4A级景区，完成周口关帝庙国家3A级景区创建工作。高度重视平粮台遗址、女娲城、吉鸿昌纪念馆、叶氏庄园等人文景观和历史文化遗存的保护修缮工作，加快周口植物园建设，发展观光农业，综合开发旅游精品。加大旅游宣传力度，面向全国和部分国家及地区，搞好宣传与推介。积极开发旅游相关产品，围绕吃住行，拉长产业链，促进交通、餐饮、住宿、娱乐、购物等相关产业发展，推动我市旅游产业跃上一个新台阶。

　　(五)扎实推进财税金融工作，提高对经济社会发展的支持能力进一步完善财税职能，构建公共财政体系。发挥金融支持作用，扩充信贷总量，加大财税金融对经济的支持力度。加强财税工作。坚持依法治税，实行综合治税，强化税收征管，做到应收尽收。优化收入结构，提高税收收入占一般预算收入的比重。大力培植和发展支柱财源、税源，把经济发展和招商引资成果体现在财政增收上，促进税收与经济协调增长。加强和规范非税收入管理，严格执行“收支两条线”规定，认真开展行政事业单位国有资产清查，增强政府宏观调控能力。牢固树立过紧日子的思想，厉行节约，勤俭办一切事情。进一步优化支出结构，突出重点，大力压缩会议费、招待费等一般性支出，确保工资改革和津贴补贴政策落实，确保党政机关运转和法定支出需要，确保城乡低保等社会保障投入。深化财政改革，扩大国库集中收付、政府采购和政府投资评审范围，促进财政科学化、规范化、精细化管理。强化财政监督，加强对重点领域和重点项目的监督检查，提高财政资金使用规范性、安全性和有效性。支持金融部门改革与发展。培植地方金融资源，加快城市信用社改组为商业银行步伐，深化农村信用社改革，积极发展合作金融组织。推动邮政储蓄小额质押贷款业务开展，支持发展保险业和证券业。加强信用担保体系和信用制度建设，积极解决中小企业融资难问题。搞好银企、银农协作，鼓励各金融机构为周口发展注入更多的资金。严惩逃废金融债务行为，优化金融生态环境。

　　(六)扎实推进改革开放，增强经济发展活力坚持深化改革和实施开放带动战略，全面扩大对外开放和区域合作，形成更具活力、更加开放的体制机制。

　　全面推进各项改革。深化国有流通企业改革。加快商贸、物资等商贸流通企业改革，不断增强发展活力。加快人事制度和事业单位改革。转换事业单位用人机制，完善聘用和岗位管理制度。加强医疗卫生体制改革，规范医疗服务行为。完成水利、交通体制改革。落实教育经费保障机制，推进农村义务教育改革。实施农村公益事业“一事一议”筹资筹劳办法。开展清理化解乡村债务试点工作。

　　大力发展非公有制经济。完善和落实政策措施，着力创造各类所有制企业平等竞争、一视同仁的法制环境、政策环境和市场环境。鼓励和支持非公有资本进入基础设施、垄断行业、公用事业及法律法规未禁止的其他行业和领域，在税费、用地、外贸、劳动保障和财政性扶持资金等方面，全面落实公平待遇。充分发挥工商联、各级商会、行业协会的服务作用，鼓励非公有资本参与国有和集体企业的改制重组，支持有条件的非公有制企业做强做大。切实保护私有财产权和非公有制企业的合法权益。

　　全面开创招商引资新局面。进一步完善新形势下加快招商引资的政策措施，不断创新招商引资工作方式和方法，突出专业招商、以商招商、驻地招商、协作招商、委托招商、网上招商等形式，努力做到“五个提高”：提高招商引资质量，特别是大项目在招商项目中的比重；提高工业项目在招商项目中的比重；提高科技含量高、附加值高、国家鼓励发展的项目在招商项目中的比重；提高签约项目的履约率和资金到位率；提高招商引资项目开工率、建成率和投产率。继续加大各县市区和市直有关部门招商引资力度，力争今年实际利用外资7000万美元以上。立足淮海经济区，密切关注珠三角、长三角、京津唐、环渤海等发达地区的产业转移情况，构建产业转移承接平台。加强产业投向引导，推出一批优质资产、优势项目，实现引资领域向三次产业全面拓展。

　　进一步扩大对外开放。充分发挥周口海关平台作用，吸引周边地区的商品从周口通关，促进我市现有企业转型升级。突出抓好莲花味精、鞋城皮革等20家重点出口企业，积极开拓国际市场，力争完成进出口3亿美元的目标。以具有地方特色的加工业为重点，努力扶持一批外向型优势企业和产品。

　　改善和优化发展环境。以政务环境建设为重点，把“亲商、安商、富商”措施落实到位。公平对待，一视同仁，兑现政策，履行承诺，营造诚信透明的政策环境；加强各级行政服务中心建设，把行政许可、行政审批和便民利民服务项目全部纳入中心办理，减少环节，集中审批，限时办结，一站服务，营造高效便捷的办事环境；多放少限，多帮少卡，多奖少罚，多予少取，营造开明有序的商务环境。继续开展政风行风评议活动，对以权谋私、损害企业和投资商及群众利益的行为，坚决从严从快查处，以硬手段整治软环境，务求取得明显成效。

　　(七)扎实推进各项社会事业发展，努力构建和谐周口坚持以人为本，把群众利益放在首位，解决好人民群众最关心、最直接、最现实的利益问题。全面推进各项社会事业。深入实施人才强市战略，建立健全吸引人才、留住人才的长效机制。坚持把教育摆在优先发展的战略地位，加大教育投入，提高农村中小学公用经费保障水平，解决市区中小学大班额问题。巩固九年义务教育成果，稳步发展高中阶段教育，大力发展职业教育，提高高等教育质量。规范学校收费行为，坚决治理教育乱收费。加强教师队伍建设，为教育发展提供师资保障。加快发展文化、卫生、体育、广播电视事业。切实加强文化市场监督管理，规范网吧、音像、娱乐业经营行为。深化城镇医疗卫生体系改革，积极推进社区卫生服务工作开展。建立健全公共卫生和医疗服务体系，抓好市中心医院病房大楼、市中医院门诊病房综合楼等续建项目。深入开展全民健身活动，大力发展体育事业。巩固广播电视“村村通”成果，提高广播电视覆盖率。认真贯彻执行严格的土地保护政策，确保土地尤其是基本农田数量不减少、质量不降低。继续推进土地综合整治，盘活存量建设用地，加强土地节约集约利用。高度重视就业和社会保障工作，关注农村留守老人生活和儿童教育，大力发展以扶老、助残、救孤、济困为重点的社会福利和慈善事业。做好民族工作，进一步巩固和发展平等、和谐、友好的民族关系，促进各民族共同发展繁荣、共同团结进步。全面贯彻党的宗教政策，依法管理宗教事务，引导宗教与社会主义社会相适应。继续做好审计、统计、物价、气象、地震、人防、外事、侨务、史志等工作，促进社会事业全面发展。严格计划生育管理工作。认真落实人口与计划生育目标责任制和一票否决权制，强化对流动人口、下岗职工和无业人群的计划生育管理，继续实施农村部分计划生育家庭奖励扶持政策，确保人口自然增长率低于全省平均水平。优化出生人口结构，提高出生人口素质，统筹解决出生人口性别比偏高等问题。加强精神文明和民主法制建设。深化爱国主义、集体主义、社会主义教育，广泛开展社会公德、职业道德、家庭美德教育，加强未成年人思想道德建设和大学生思想政治教育，在全社会树立知荣辱、讲正气、树新风、促和谐的新风尚。继续开展文明行业、文明单位、文明村户创建和“双百”评选活动。深入开展文化、科技、卫生“三下乡”和科教、文体、法律、卫生进社区活动，丰富群众文化生活，提高全民科学文化素质。坚持依法治市，继续开展普法教育，提高全民法律意识。自觉接受人大及其常委会的法律监督、工作监督和政协的民主监督，严格执行人大及其常委会的决议、决定，支持人大代表依法行使职权，认真办理人大代表建议和政协委员提案。广泛听取各民主党派、工商联和无党派人士的意见和建议，充分发挥工会、共青团、妇联等人民团体的桥梁纽带作用。加强法律援助，依法保障公民特别是妇女、未成年人和残疾人的合法权益。积极支持国防和军队建设。加强国防教育，提高全民国防意识。满腔热情地关心、支持驻周解放军、武警消防部队和民兵预备役建设，做好军队转业干部、退伍军人和随军家属安置工作。深入开展拥军优属和军民共建活动，进一步密切军政军民关系。切实做好社会稳定工作。高度重视公共安全，完善应急管理体系，有效应对各种风险。严格落实安全生产责任制，认真做好消防工作，对存在重大安全隐患的生产经营场所，坚决予以关停整顿。大力整顿和规范市场经济秩序，加大打击制假售假专项工作力度，切实加强食品药品安全监管，保障人民群众身体健康。加强社会治安综合治理，完善社会治安防控体系，继续开展打击“两抢一盗”专项斗争，依法严厉打击各种违法犯罪活动。认真做好信访工作，建立完善矛盾调处和社会利益协调机制，从源头上预防和减少矛盾发生。

　　四、加强政府自身建设

　　新一届人民政府肩负着发展的使命，承载着人民的重托。为适应新形势新任务要求，我们要进一步加强政府自身建设，努力做到为人民服务、对人民负责、受人民监督、让人民满意。转变职能，建设效能政府。按照精简、统一、效能的原则和决策、执行、监督相协调的要求，进一步清理、减少行政审批事项，规范行政审批行为。积极转变政府管理经济方式，搞好经济调节和市场监管，着力为市场主体创造公平竞争环境和提供良好服务；更加注重履行社会管理和公共服务职能，把政府工作的着力点更多地放在发展社会事业和构建和谐社会上。积极整合行政资源，有效降低行政成本。加强行政效能监察，严格执行问责制，提高政府执行力，确保圆满完成各项目标任务。依法行政，建设法治政府。加强政府法制建设，加快建立权责明确、行为规范的行政执法体制，保证各级行政管理机关及其工作人员严格按照法定权限和程序履行职责。依法规范行政执法主体，解决行政执法职责交叉、多头执法、效率不高等问题。推行行政执法责任制、评议考核制和过错责任追究制，确保行政执法人员严格执法、公正执法、文明执法。强化行政监督，做好行政复议工作，切实做到有权必有责、用权受监督、侵权须赔偿、违法受追究。认真实施《公务员法》，加强公务员教育、管理和监督，建设高素质的公务员队伍。

　　公开政务，建设阳光政府。全面推行政务公开，切实保障广大人民群众的知情权、参与权和监督权。完善政府新闻发言人制度。推行决策公示、听证和专家论证、技术咨询、邀请人民群众旁听有关会议等制度，扩大政务公开范围，拓宽政务公开渠道，增强行政透明度，提高政府公信力。坚持以法律为基础，全面提升政府诚信，重点强化制度信用、政策信用和程序信用，树立信守承诺、务实高效的诚信政府形象。加快电子政务建设步伐，推进政府信息资源的整合与利用，构建开放式政府信息平台，为公众提供便捷的电子化服务。进一步办好市长热线和市长信箱，认真倾听群众呼声，不断改进政府工作。强化服务，建设民本政府。把实现好、维护好、发展好人民群众的根本利益作为工作的出发点和立足点，把执政为民的要求融入到思想感情里，体现在政策措施中，落实到具体工作上。深怀爱民之心，恪守为民之责，善谋富民之策，多办利民之事。深入基层，深入群众，倾听民声，尊重民意，对发展中的热点难点问题，一个一个地研究，一个一个地解决。建立健全责任考评机制，把评定政府工作绩效的权力交给人民群众，把敢负责作为政治品质的集中体现，把创业绩作为能力才干的鉴别标准，激励政府工作人员强化宗旨观念，提高服务水平。从严治政，建设廉洁政府。认真贯彻落实中纪委七次全会精神，大力倡导八种良好风气，全面加强思想作风、学风、工作作风、领导作风、干部生活作风建设。坚持从严治政，筑牢拒腐防变的思想道德防线。健全教育、制度、监督并重的惩治和预防腐败体系，建立思想道德教育长效机制、反腐倡廉制度体系、权力运行监控机制。认真执行廉洁自律各项规定，全面落实廉政建设责任制，深入开展反腐纠风工作。充分发挥监察、审计等监管职能，全面推行领导干部经济责任审计制度，严格项目招投标、土地“招拍挂”、国有资产转让和罚缴分离等制度，严防违法违纪行为发生，使政府权力在制度轨道中运行，树立清正廉洁的政府形象。

　　各位代表，周口历史悠久，人杰地灵。先贤圣哲、名人巨擘灿若群星。数不清的历史人物，展示了周口人的风采。这是一方干事创业的热土，这是一个有着无限发展潜力和广阔发展前景的地方。实现追赶型跨越式发展，是历史赋予我们的庄严使命，是全市1070万人民的殷切期盼。尽管前进的道路不会平坦，但任何困难都动摇不了我们加快发展的决心和信心！

　　各位代表，形势催人奋进，事业任重道远。我们的使命崇高而神圣，我们的责任重大而光荣。让我们在中共周口市委的坚强领导下，在市人大、市政协的监督支持下，紧紧依靠全市人民，以更加饱满的热情、昂扬的斗志、创新的精神、务实的作风，团结拼搏，开拓进取，努力实现我市追赶型跨越式发展，为建设一个繁荣富裕、文明开放、稳定和谐的新周口而努力奋斗！
